# Supplementary figures and images for: Discovery of gene regulatory elements through a new bioinformatics analysis of haploid genetic screens
Source: PLoS One. 2019 Jan 29;14(1):e0198463. doi: 10.1371/journal.pone.0198463 (PMC6350973; doi:10.1371/journal.pone.0198463)

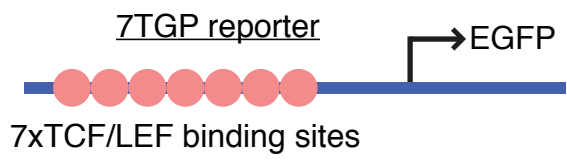

Supplement: S1 Fig — HAP1-7TGP cells harbor an enhanced green fluorescent protein (EGFP) reporter driven by an established WNT-responsive element containing seven TCF/LEF-binding sites upstream of a minimal promoter. (PDF) [file pone.0198463.s001.pdf]

**A**

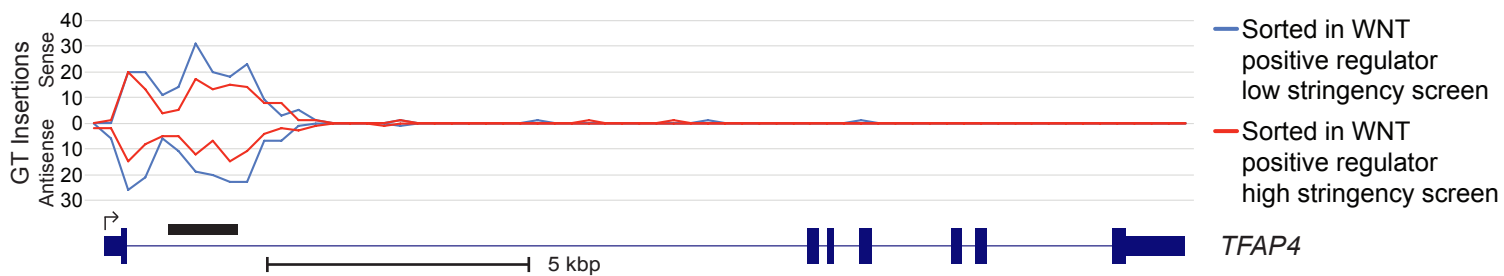

**B**

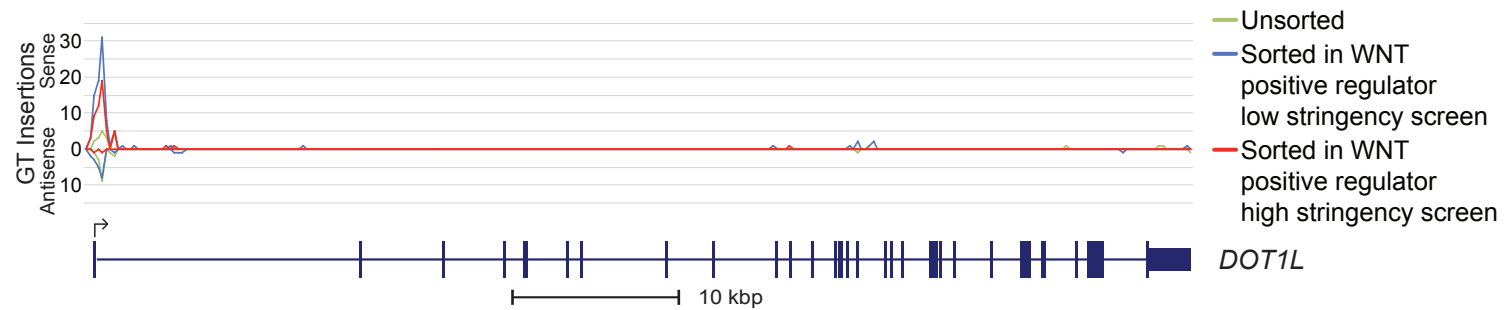

Supplement: S2 Fig — (A) The histogram indicates the number and orientation of insertions mapped to TFAP4 in the sorted cell populations from the WNT positive regulator low stringency and high stringency screens. See legend to Fig 4A for details. (B) The histogram indicates the number and orientation of insertions mapped to DOT1L (Chromosome 19, 2163750–2232749 bp) in unsorted cells and in the sorted cell populations from the WNT positive regulator low stringency and high stringency screens. The pattern of GT insertions seen in DOT1L, predominantly enriched for sense insertions in the first intron, differs from the observed enrichment for both sense and antisense insertions seen in the first intron of TFAP4. (PDF) [file pone.0198463.s002.pdf]

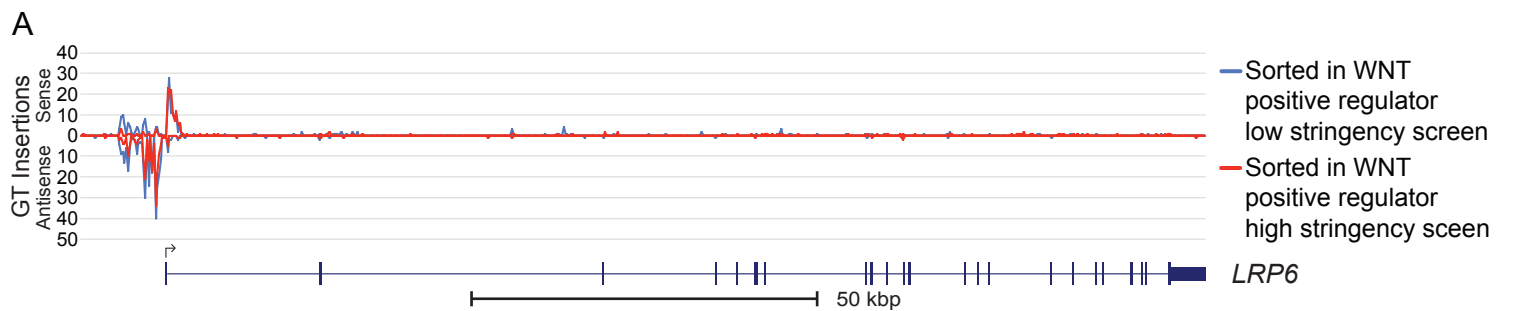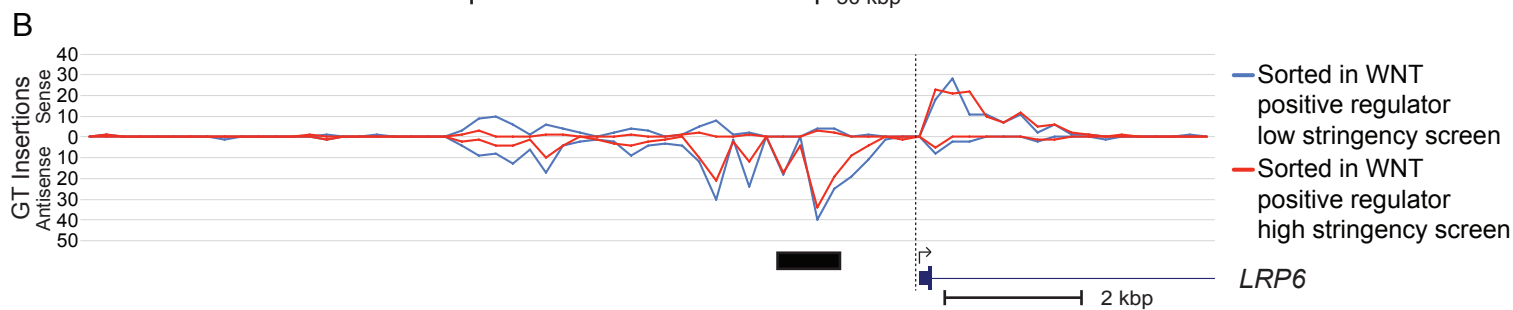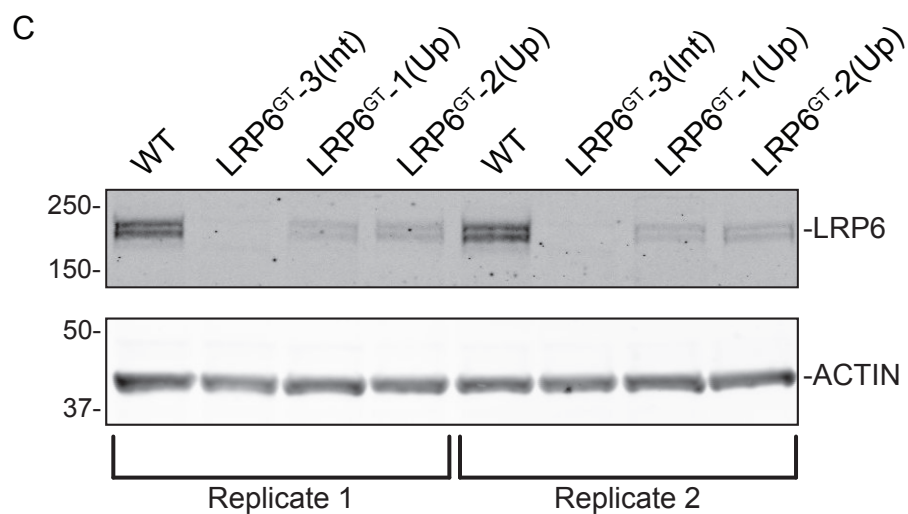

Supplement: S3 Fig — (A) The histogram indicates the number and orientation of insertions mapped to LRP6 and to the region ~12.5 kbp upstream of the TSS in the sorted cell populations from the WNT positive regulator low stringency and high stringency screens. See legend to Fig 5A for details. (B) The histogram shows an expanded view of the 5’ end of LRP6 and the region ~12.5 kbp upstream of the TSS (left of the vertical dotted line), with traces for GT insertions mapped in the sorted cell populations from the WNT positive regulator low stringency and high stringency screens. See legend to Fig 5B for details. (C) Immunoblot analysis of LRP6. The top and bottom parts of the same membrane were probed for LRP6 and ACTIN (loading control), respectively. The cell lines from which the samples were prepared and loaded in duplicate are indicated above the blots. Molecular weight standards in kDa are indicated to the left of each panel. (PDF) [file pone.0198463.s003.pdf]
